# A Novel Immunological Assay for Hepcidin Quantification in Human Serum

**DOI:** 10.1371/journal.pone.0004581

**Published:** 2009-02-24

**Authors:** Vasiliki Koliaraki, Martha Marinou, Theodoros P. Vassilakopoulos, Eustathios Vavourakis, Emmanuel Tsochatzis, Gerassimos A. Pangalis, George Papatheodoridis, Alexandra Stamoulakatou, Dorine W. Swinkels, George Papanikolaou, Avgi Mamalaki

**Affiliations:** 1 Laboratory of Molecular Biology and Immunobiotechnology, Department of Biochemistry, Hellenic Pasteur Institute, Athens, Greece; 2 1st Department of Internal Medicine and Department of Hematology, Athens University Medical School, Athens, Greece; 3 Hematology Laboratory, Aghia Sophia Children's Hospital, Athens, Greece; 4 2nd Department of Internal Medicine, Athens University Medical School, Hippokration General Hospital of Athens, Athens, Greece; 5 Department of Clinical Chemistry, Radboud University Nijmegen Medical Centre, Nijmegen, the Netherlands; Cleveland Clinic, United States of America

## Abstract

**Background:**

Hepcidin is a 25-aminoacid cysteine-rich iron regulating peptide. Increased hepcidin concentrations lead to iron sequestration in macrophages, contributing to the pathogenesis of anaemia of chronic disease whereas decreased hepcidin is observed in iron deficiency and primary iron overload diseases such as hereditary hemochromatosis. Hepcidin quantification in human blood or urine may provide further insights for the pathogenesis of disorders of iron homeostasis and might prove a valuable tool for clinicians for the differential diagnosis of anaemia. This study describes a specific and non-operator demanding immunoassay for hepcidin quantification in human sera.

**Methods and Findings:**

An ELISA assay was developed for measuring hepcidin serum concentration using a recombinant hepcidin25-His peptide and a polyclonal antibody against this peptide, which was able to identify native hepcidin. The ELISA assay had a detection range of 10–1500 µg/L and a detection limit of 5.4 µg/L. The intra- and interassay coefficients of variance ranged from 8–15% and 5–16%, respectively. Mean linearity and recovery were 101% and 107%, respectively. Mean hepcidin levels were significantly lower in 7 patients with juvenile hemochromatosis (12.8 µg/L) and 10 patients with iron deficiency anemia (15.7 µg/L) and higher in 7 patients with Hodgkin lymphoma (116.7 µg/L) compared to 32 age-matched healthy controls (42.7 µg/L).

**Conclusions:**

We describe a new simple ELISA assay for measuring hepcidin in human serum with sufficient accuracy and reproducibility.

## Introduction

Hepcidin is a 25-aminoacid cysteine-rich peptide, present in human serum and urine [1], [2]. It is synthesized predominantly by hepatocytes as an 84-aminoacid precursor protein and its mature form is released in circulation [3]. Hepcidin acts by binding to ferroportin (FPN1), the only known cell iron exporter [4], inducing its internalization and subsequent degradation in the cytoplasm [5]. In systemic level, hepcidin upregulation results in inhibition of iron absorption from intestinal enterocytes and iron recycling from macrophages [5], [6]. Hepcidin expression is up-regulated by iron and inflammation and down-regulated by anaemia and hypoxia [7].

Studies in humans have highlighted the crucial role of hepcidin in the regulation of iron homeostasis. Homozygous mutations of the gene encoding for hepcidin (*HAMP*) lead to rare cases of juvenile hemochromatosis [8]. Furthermore, hepcidin in hemojuvelin (HJV) associated juvenile hemochromatosis is downregulated [8], [9]. Low or relatively low hepcidin levels, for the degree of iron load, have been also found in patients with TFR2 hemochromatosis and secondary iron overload diseases such as thalassaemia intermedia, thalasaemia major and chronic hepatitis C [10]–[12]. In contrast, increased hepcidin levels have been reported in end-stage renal disease and inflammation [13], [14].

Several methods for hepcidin quantification in human serum and urine have been reported. There are two antibody-based techniques, a dotblot assay, which has been used to measure hepcidin in urine and is considered semi-quantitative [15], [16], and a commercially available ELISA assay measuring serum prohepcidin. The diagnostic utility of the latter is controversial, since prohepcidin represents a processing intermediate rather than a biologically significant form [17], [18].

Lately there has been a growing interest in the development of quantification techniques based on mass spectrometry (matrix-assisted laser desorption ionization (MALDI-), surface enhanced laser desorption/ionisation time-of flight mass spectrometry (SELDI-TOF MS), or liquid chromatography tandem mass spectrometry (LC-MS/MS)) that show promising results [8], [13], [19], [20], [21], [22], [23]. However, some of these approaches did not use internal standard for the quantification of hepcidin and are considered semi-quantitative and moreover they require specialized equipment that are not widely accessible [8]. Therefore, the development of a facile ELISA assay remains an unmet need and a potentially valuable tool for research and diagnostic applications, although the presence of other hepcidin iso-forms in human samples, such as hepcidin20 and hepcidin22, might be a limitation. Recently, a competitive ELISA method for quantification of hepcidin was published using functional synthetic peptides [24]. In this study, we sought to develop an alternative ELISA method for hepcidin quantification in human serum, using a recombinant functional hepcidin25-His peptide, which offers the advantage of a functional tagged molecule produced at high yield and a polyclonal antibody against this peptide, which can identify native hepcidin.

## Results

### Production and characterization of a polyclonal antibody against hepcidin

Recombinant hepcidin25-His was expressed in yeast *P. pastoris* and the biological activity of the isolated monomer was tested according to Koliaraki et al [25]. Recombinant hepcidin25-His was used for the immunisation of rabbits and the polyclonal antiserum was extensively purified with affinity chromatography, as described in Materials and Methods. Western blot analysis showed that the recombinant hepcidin-25His was detected by the polyclonal antibody, whereas another peptide of approximately the same size, bearing a Myc-His tag and produced in the same way in *P. pastoris* (namely negative peptide) [25] was not detected (data not shown).

**Figure 1:**
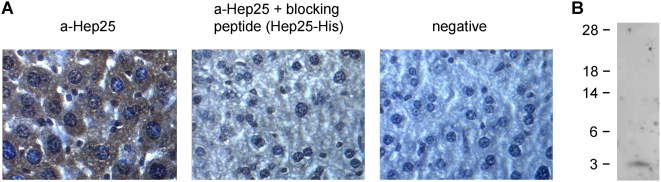
Specificity of the polyclonal antibody against native hepcidin. (A) Immunohistochemical staining of liver tissue sections using the polyclonal antibody against hepcidin25-His (a-Hep25). Secondary anti-rabbit antibody was used as negative control (negative). The specificity of the polyclonal antibody was verified after reduction of the signal following preincubation with hepcidin25-His. (B) Western blot analysis of serum proteins less than 30 kDa. 10 ml of serum was filtered through a 30 kDa filter, and the filtrate was precipitated with 25% TCA. Precipitated proteins were subjected to electrophoresis on a 4–12% Nu-PAGE gel, followed by Western blot using the polyclonal antibody against hepcidin25-His.

In order to determine its binding activity against native hepcidin we first performed immunohistochemistry on paraffin embedded mouse liver sections. The antibody showed a strong cytoplasmic staining in hepatocytes that was reduced after preincubation with hepcidin25-His (Fig. 1A). Furthermore, the specificity of the antibody against the native peptide in serum was shown by Western Blot analysis of TCA-precipitated proteins less than 30 kDa from 10 ml of serum. A single band at 3 kDa was detected (Fig. 1B). This signal was abolished when the polyclonal antibody was preincubated with the recombinant peptide hepcidin25-His (data not shown).

### Development and characterization of an ELISA assay for serum hepcidin measurement

The recombinant peptide and the polyclonal antibody against it were used for the development of an immunological assay for the quantification of hepcidin in human serum. After determining the optimal concentration of antigen and antibody according to Crowther [26], we proceeded to the analytical characterisation of our ELISA system.

**Figure 2:**
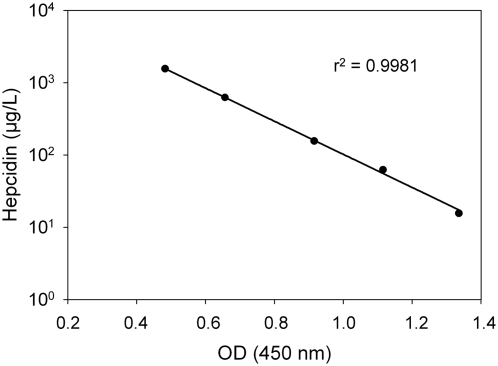
Representative calibration curve for recombinant hepcidin25-His. The range of the assay is 10–1500 µg/L.

Our competition ELISA produces a typical calibration curve for the recombinant hepcidin25-His (Fig. 2). The analytical limit of detection of the ELISA assay, defined as the concentration corresponding to the mean signal+3 SD of 10 replicates of the zero calibrator was 5.4 µg/L. The measurement range was 10–1500 µg/L. For the statistical analysis of the reproducibility, linearity and recovery of the hepcidin ELISA assay, we used 3 serum samples ranging from low (22 µg/L) to high (150 µg/L) concentrations chosen from 32 normal sera tested. The intra-assay coefficients of variance (CVs) were 8–15% as evaluated by assaying 12 replicates of each sample in a single assay (Table 1). The inter-assay CVs were 5–16% as evaluated by 7 subsequent measurements of the test samples (Table 2). Intra- and inter-assay CV for the standard curve was 2.8% and 8.5%, respectively. Analytical recovery was studied by adding the calibrator at 7.5, 30 and 75 µg/L in each serum sample and was found to range from 99–115% with a mean recovery index of 107% (Table 3). Mean linearity was estimated at 101% after measuring 3 serial dilutions (1∶2, 1∶4, 1∶8) of the 3 serum samples (Table 4).

**Table 1:** Intra-assay variation.

	N	Mean±SD (µg/L)	CV%
***Serum 1***	12	23.25±3.6	15.4
***Serum 2***	12	57±5.7	9.94
***Serum 3***	12	166.6±13	7.83

**Table 2:** Inter-assay variation.

	N	Mean±SD (µg/L)	CV%
***Serum 1***	7	25.6±3.9	15.33
***Serum 2***	7	57.6±2.5	4.32
***Serum 3***	7	184.4±16.4	8.88

**Table 3:** Recovery of calibrator added to human serum samples.

	Endogenous hepcidin (µg/L)	Calibrator added (µg/L)	Expected (µg/L)	Observed (µg/L)	Percent Recovery^a^
***Serum 1***	28.5				
		7.5	36.02	40.86	113.4
		30	58.52	58.35	99.7
		75	103.52	119.26	115.2
***Serum 2***	59.7				
		7.5	67.2	70.95	105.6
		30	89.7	89.05	99.3
		75	134.7	153.2	113.75
***Serum 3***	177				
		7.5	184.5	189.37	102.6
		30	207	205.77	99.4
		75	252	283.73	112.6

a (Observed concentration/Expected concentration)×100.

**Table 4:** Dilution linearity of ELISA.

	Hepcidin (µg/L)	Dilution factor	Expected (µg/L)	Observed (µg/L)	Percent Recovery^a^
***Serum 1***	23.34	1	23.34		
		2	11.67	11.6	99.4
		4	5.83	5.97	102.4
		8	2.92	3.05	104.6
***Serum 2***	57.73	1	57.73		
		2	28.86	28.45	98.5
		4	14.43	13.77	95.4
		8	7.22	7.25	100.5
***Serum 3***	164.76	1	164.76		
		2	82.38	82.95	100.7
		4	41.19	42.67	103.6
		8	20.59	21.27	103.3

a (Observed concentration/Expected concentration)×100.

### Clinical evaluation in iron disorders

In order to determine whether our assay was providing biologically meaningful measurements, we tested serum samples from patients with anticipated low hepcidin levels (HJV associated juvenile hemochromatosis and iron deficiency anemia) [4], [8], [16], [27], compared to healthy controls, as well as from patients with anticipated high hepcidin levels (Hodgkin's lymphoma with B-symptoms) [15], [28], [29], [30].

**Figure 3:**
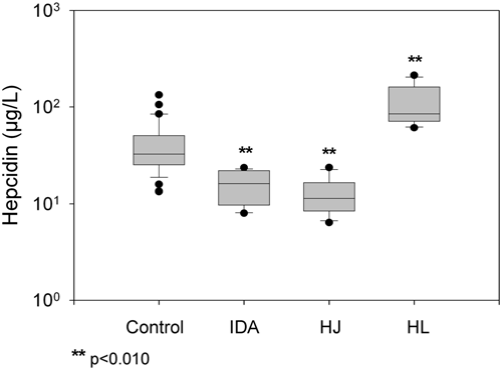
Hepcidin serum concentration in healthy controls (control), patients with iron deficiency anemia (IDA), juvenile haemochromatosis (JH) and Hodgkin's lymphoma (HL). Box plots show the 25th and 75th percentile with median value for each group. Minimum and maximum values are also depicted. Significant difference compared to control is indicated by asterisk (**p<0.010).

Mean hepcidin concentration was significantly lower in 10 patients with iron deficiency anemia (15.7 µg/L, p<0.010) and 7 patients with juvenile hemochromatosis (12.8 µg/L, p<0.010) and significantly higher in 7 untreated patients with Hodgkin's lymphoma and B-symptoms (116.7 µg/L, p<0.010), compared to age-matched healthy controls (42.7 µg/L), as shown in Fig. 3.

**Figure 4:**
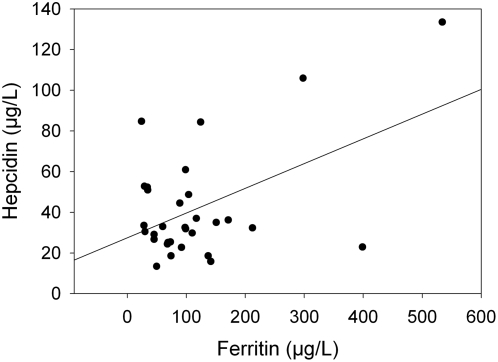
Correlation between serum hepcidin and ferritin in healthy controls. Hepcidin values measured by our ELISA assay correlate significantly with ferritin levels. Pearson correlation: 0.474 (p = 0.006).

Hepcidin levels in 32 healthy controls ranged from 13.4 to 133.5 µg/L, and correlated with serum ferritin (Pearson correlation: 0.474, p = 0.006) (Fig. 4).

### Correlation with a mass-spec method

**Figure 5:**
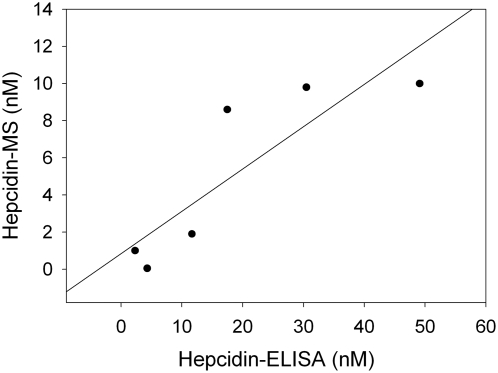
Correlation between serum hepcidin measured by our ELISA assay and by the SELDI-TOF-MS method. Hepcidin values of 6 serum samples, measured by the two methods correlate significantly. Pearson correlation: 0.863 (p = 0.027).

In order to better evaluate our assay we performed a comparison with an already established SELDI-TOF-MS method [22]. We used 6 serum samples, two of each patient category (control, low and high hepcidin concentrations). The results from the MS assay are expressed in nM with the use of the equation: (sample 2789 m/z peak intensity)×10 nM/(hepc24 spiked sample 2673 m/z peak intensity– non spiked sample 2673 m/z peak intensity). Hepcidin values, measured by our ELISA assay were also converted to nM. Pearson correlation showed a significant correlation between our assay and the mass-spec method (Pearson correlation: 0.863, p = 0.027) (Fig. 5).

## Discussion

The present study describes a new, biologically significant immunological assay for hepcidin quantification in human serum, based on the use of a recombinant hepcidin peptide and a polyclonal antibody. The recombinant hepcidin25-His was previously produced in *P. pastoris*, a system that offers the advantage of soluble expression at high yield and without the need of any renaturation procedure. The recombinant hepcidin25-His peptide was produced in a near native conformation with all 8 cysteines taking part in disulfide bond formation, as suggested by mass-spectrometry, and was found to be bactericidal and active in modulating cellular iron homeostasis [25]. The recombinant peptide was shown to mimic biological activity of native hepcidin as prior studies illustrated its ability to bind ferroportin causing its internalisation and promoting an increase in the levels of the LIP and a decrease in the expression levels of transferrin receptor 1 in macrophages [25].

So far the antibody based methods for hepcidin quantification in human serum are either semi-quantitative [15], [16] or measure the prohormone whose correlation to the levels of hepcidin25 remains controversial [17], [18]. Specifically, prohepcidin does not respond to physiological stimuli, such as inflammation [30] or iron absorption and it does not correlate to iron parameters or serum hepcidin [31], [32]. As for the mass spectrometry based techniques [8], [13], [19], [20], [21], [22], [23] although they are continuously being improved their accessibility is still limited. The development of a simple immunoassay for hepcidin quantification is expected to increase availability of analysis of biological specimens in clinical laboratories, providing information for the role of hepcidin in health and disease.

To this direction, we produced a polyclonal antibody against the recombinant peptide, which was also able to recognize native hepcidin. Specifically, this antibody showed a strong cytoplasmic staining in hepatocytes and recognized a single band at approximately 3 kDa in human serum. Both signals were abolished after preicubation of the antibody with recombinant hepcidin25-His. Furthermore, the Western blot of human serum led to the conclusion that the antibody was specific for the mature hepcidin peptide found in serum, while propeptides were not detected, as the immature peptide should not normally be secreted in the serum [3]. However, we cannot still exclude the possibility of its binding to the truncated 20aa or 22aa form, as with current immunochemical methods methods it is difficult to detect such a band. Subsequently, we used this antibody to develop a competition ELISA for measuring serum hepcidin concentration. After examining the analytical characteristics of this assay, which were found satisfactory, we proceeded in determining the clinical value of it by measuring hepcidin levels in patients with several iron disorders. We used patients with iron deficiency anemia, HJV-associated juvenile hemochromatosis and Hodgkin's lymphoma, manifesting B-symptoms and compared them with healthy controls. It is of note that in healthy controls hepcidin values, which ranged from 13.5 to 133.5 µg/L, correlated well with ferritin values. This implies a high association with iron stores, which is important for the verification of the biological significance of the assay. In iron deficiency anemia hepcidin is normally reduced in order to increase iron absorption and recycling [4]. In juvenile hemochromatosis, mutations in HJV gene have as a result the decrease of hepcidin expression and the subsequent iron overload [27]. We found significantly lower hepcidin levels in both iron deficiency anemia and juvenile hemochromatosis, as expected. Hodgkin's lymphoma is clinically characterized by symptoms resembling those of chronic infectious diseases and levels of interleukin-6 are high in untreated patients [28]. Since interleukin-6 directly induces hepcidin expression, high hepcidin levels were expected, as we found in these patients [15], [29], [30]. Moreover, in order to better evaluate our results we performed a comparison between our competition ELISA and a SELDI-TOF-MS technique, which is well established [22], and found them to correlate significantly. It is of note that absolute hepcidin values vary between the two methods, which is probably due to the different quantification of the two standards. These results allow us to conclude that this new ELISA system can effectively quantify hepcidin in human serum.

Recently another competitive ELISA method for quantification of hepcidin in human fluids was also published, reporting reproducible measurements in serum and urine [24]. This assay was performed by using a functional biotinylated synthetic peptide as a tracer (Intrinsic LifeSciences, La Jolla, CA), and another one for the construction of the standard curve (Bachem, King of Prussia, PA). Our method is based on a different principle from the one described by this recent work and moreover it utilizes a functional recombinant peptide. The growing interest in this field and the new or improved methods that have been published recently render the universal standardization necessary, in order to gain meaningful results from hepcidin measurements.

In conclusion, we have developed an ELISA assay capable of measuring hepcidin in human serum samples at a range of 10–1500 µg/L with acceptable imprecision, linearity, recovery and specificity. This assay is easy to apply and utilizes a small amount of unprocessed serum, bypassing time-consuming preanalytical procedures. A reliable and easy to apply hepcidin quantification method could prove a valuable tool for diagnostic applications, as hepcidin levels may be used for the differential diagnosis of several disorders including iron deficiency, functional iron deficiency in patients with renal anemia, anemia of chronic disease and iron overload disorders.

## Materials and Methods

### Subjects

This study included 32 healthy controls, 7 patients with HJV associated juvenile heamochromatosis, 10 with iron deficiency anemia and 7 with Hodgkin's Lymphoma manifesting B symptoms. The study was approved by the ethics committees of the participating institutions. Informed consent was obtained from all patients and controls and study procedures were conducted in accordance with to the Declaration of Helsinki.

### Expression and purification of recombinant hepcidin25-His

For the development of the assay, we used the previously reported recombinant hepcidin25-His in its monomeric form. This is expressed in yeast *P. pastoris* and retains the functional and structural properties of the native form [25]. Briefly, a *P. pastoris* clone expressing hepcidin25 with a His-tag at its C-terminal was grown for 24 h at 30°C and then induced for 3 days by methanol 0.5%, as described in [25]. Culture supernantant was concentrated and dialysed with the use of a TFF Prep scale Ultrafiltration system equipped with a 1 kDa filter (Millipore, Bedford, MA). The concentrate was first purified using Ni^2^-NTA metal affinity chromatography according to the manufacturer's instructions (Qiagen, Hilden, Germany) and then by size exclusion chromatography with a Peptide Superdex column (Amersham Biosciences, Munich, Germany) in order to isolate the monomer.

### Immunization procedure

100 µg of the recombinant hepcidin25-His diluted into 0.4 mL in PBS were mixed with 0.4 mL of Complete Freund's adjuvant (Sigma, St. Louis, MO), and injected into New Zealand white rabbits subcutaneously. Three boosts in Incomplete Freund's adjuvant were performed every 4 weeks following the primary immunization. A sample of pre-immune serum was taken from the ear vein before the first injection. The test bleeding was carried out 10 days after the last boost immunization from the ear vein. The serum was tested for antibody activity with ELISA assay (data not shown).

### Purification of the polyclonal antibody

Polyclonal antiserum was extensively purified by affinity chromatography. Briefly, total IgGs were first precipitated with 33% ammonium sulfate and the IgG fraction was dialyzed against PBS [33]. The elimination of the His-tag specific antibodies from the IgG fraction was performed by repeated purifications on a column with CNBr-activated Sepharose beads (GE Healthcare, Uppsala, Sweden) coupled with the 6×HIS peptide (Covance, Princeton, NJ), whereas the specific antibodies against hepcidin were isolated by affinity chromatography with CNBr-activated Sepharose beads coupled with the hepcidin25-His peptide.

### Immunohistochemistry

Liver tissues from normal mice (C57BL/6) were deparaffinized two times in xylene for 5 min and dehydrated in dilutions of ethanol (100%, 95%, 70%) for 2 min each. Endogenous peroxidase activity was quenched with 3% hydrogen peroxide for 30 min, followed by immersion in tap water for 5 min. Antigen retrieval was accomplished by immersing and heating the slides in 10 mM citrate buffer, pH 6.0 three times in a microwave, for 5 min each, after which they were allowed to cool to room temperature for 20 min. The slides were then incubated in blocking solution (3% FBS, 1%BSA, 0.05% Tween in TBS) for 30 min, followed by incubation with purified primary antibody (10 µg/ml) at 4°C for 16 h. After that they were then incubated with secondary anti-rabbit antibody conjugated with HRP (diluted 1∶100 in blocking buffer) (DakoCytomation, Carpinteria, CA) for 1 h. Visualization of the stain was accomplished after addition of 3,3′ diaminobenzidine substrate (Sigma, St Louis, MO) for 5 min. The reaction was stopped by washing with tap water and slides were counterstained with Hematoxylin (Sigma, St Louis, MO) for 1 min. They were then dehydrated in a 70%, 95% and 100% ethanol series for 2 min each, cleared by immersing in xylene twice for 5 min each, and mounted in DPX (Sigma, St Louis, MO). Secondary antibody alone was used as negative control. In addition, competition experiments were performed by pre-incubating overnight at 4°C the polyclonal antibody with hepcidin25His peptide (1 mg/ml).

### Western blot analysis

10 ml of serum were diluted three times in PBS and filtered through a 0.22 mm filter (Millipore, Bedford, MA). After addition of 10 mm DTT, serum was filtered again through a 30 kDa cut-off filter devise (Millipore, Bedford, MA) and the filtrate was precipitated using 25% TCA. The precipitated proteins were resuspended in loading buffer and subjected to SDS electrophoresis on a 4–12% NuPAGE Novex Bis/Tris gel (Invitrogen, San Diego, CA). Western blot was performed using an XCell II blot module (Invitrogen, San Diego, CA) and a Protran nitrocellulose membrane (Schleicher & Schuell, Dassel, Germany) with 0.1 mm pore size. Non-specific sites were blocked with 5% milk in PBS and the membrane was probed with rabbit anti-hepcidin25His antibody (0.5 µg/ml in blocking buffer) or rabbit anti-hepcidin25His antibody pre-incubated for 1 h at 37°C with hepcidin25His (50 µg/ml), followed by incubation with anti-rabbit secondary antibody conjugated with HRP (DakoCytomation, Carpinteria, CA). Specific signals were detected with a chemiluminescence assay kit (ECL, Amersham Biosciences, Munich, Germany).

### ELISA procedure

96-well microtiter plates (Costar, Corning, NY) were coated with hepcidin25-His diluted at 0.5 mg/L in PBS, pH 7.4, at 4°C, for 16 h. Simultaneously, the polyclonal antibody diluted at 0.33 mg/L in 3% BSA in PBS was incubated with an equal amount of the calibrator or diluted serum (8 µl in 25 µl PBS per well) for 16 h at 4°C. As calibrator we used hepcidin25-His diluted in PBS (5, 20, 50, 200, 500 µg/L). Quantification of the recombinant peptide was performed with a fluorescent quantification system (Quant-It, Qubit), according to the manufacturer's instructions (Invitrogen, San Diego, CA). Subsequently, the plates were washed twice with PBS and blocked with 100 µl of 3% BSA in PBS for 1 h at 37°C. The complexes formed were then added to the coated wells in quadruplicates and incubated for 1 h at 37°C. After 10 washes with PBS containing 0.5 ml/L Tween 20, the plates were incubated with a secondary anti-rabbit antibody conjugated with HRP (DakoCytomation, Carpinteria, CA) diluted 1∶2000 in 3% BSA in PBS for 1 h at room temperature. The plates were washed as before and visualization of the signal was accomplished after addition of 3,3′,5,5′ tetramethylbenzidin (Pierce, Rockford, IL) for 10 min at room temperature. The reaction was stopped after the addition of 0.2 N sulphuric acid and color development was measured photometrically at 450 nm with a microplate reader (Bio-rad Model 680, Biorad, Hercules, CA). Construction of the standard curve was performed with Microsoft Office Excel.

### Hepcidin measurements with SELDI-TOF-MS

For the comparison of our assay with an already existing method 6 serum samples (two from healthy controls, two from juvenile hemochromatosis and two from Hodgkin's Lymphoma) were sent to the Department of clinical chemistry, Radboud University Nijmegen Medical Centre for measuring with SELDI-TOF-MS [22]. In short, internal standard (synthetic hepcidin-24, Peptide Int., Louisville KY) was added to serum before total hepcidin was isolated from the sample as described [1]. Next, SELDI-TOF MS was used to quantify hepcidin-25.

### Statistical analysis

Statistical analysis was performed using the SPSS software (version 16.0, SPSS Inc., Chicago, IL). Difference between hepcidin concentration in controls and different groups of patients were analyzed by Mann-Whitney *U*-test. P values <0.05 were considered to be statistically significant. Inter- and intra-assay coefficients of variance were calculated in order to determine the accuracy of the assay.
